# Microbial Communities and Correlation between Microbiota and Volatile Compounds in Fermentation Starters of Chinese Sweet Rice Wine from Different Regions

**DOI:** 10.3390/foods12152932

**Published:** 2023-08-02

**Authors:** Jing Zou, Xiaohui Chen, Chenyu Wang, Yang Liu, Miao Li, Xinyuan Pan, Xuedong Chang

**Affiliations:** 1 College of Food Science and Technology, Hebei Normal University of Science and Technology, Qinhuangdao 066104, China; huisu4ever@163.com (X.C.); wangchenyu2266@163.com (C.W.); liuyang2740@163.com (Y.L.); limiao1113@163.com (M.L.); pxy18556579567@163.com (X.P.); cxdsgx@163.com (X.C.); 2 Engineering Research Center of the Ministry of Education of Chestnut Industry Technology, Qinhuangdao 066000, China

**Keywords:** Chinese sweet rice wine, volatile compounds, microbial community, metabolic function, correlation analysis

## Abstract

Chinese sweet rice wines (CSRW) are traditional, regionally distinct alcoholic beverages that are generally brewed with glutinous rice and fermentation starters. This study aimed to characterize microbial communities and volatile compounds of CSRW starters and explore correlations between them. The major volatiles in starters include 1-heptanol, 1-octanol, 2-nonanol, phenylethyl alcohol, 2-nonanone, acetophenone, and benzaldehyde. Microbiological analysis based on high-throughput sequencing (HTS) technology demonstrated that starter bacterial communities are dominated by *Weissella*, *Pediococcus*, and *Lactobacillus*, while *Saccharomycopsis* and *Rhizopus* predominate in fungal communities. Carbohydrate and amino acid metabolism are the most active metabolic pathways in starters. Spearman correlation analysis revealed that 15 important volatile compounds including alcohols, acids, aldehydes and esters were significantly positively correlated with nine microbial genera (|r| > 0.7, *p* < 0.05), including five bacterial genera (i.e., *Weissella*, *Pediococcus*, *Lactobacillus*, *Bacillus*, and *Nocardiopsis*) and four fungal genera (i.e., *Saccharomycopsis*, *Rhizopus*, *Wickerhamomyces*, and *Cyberlindnera*), spanning 19 distinct relationships and these microorganisms were considered the core functional microorganisms in CSRW starters. The most important positive correlations detected between phenylethyl alcohol and *Weissella* or *Saccharomycopsis* and between 2-nonanol and *Pediococcus*. This study can serve as a reference to guide the development of defined starter cultures for improving the aromatic quality of CSRW.

## 1. Introduction

Rice wine is popular in East and Southeast Asian countries, such as Chinese sweet rice wine and yellow rice wine, Japanese sake, Korean rice wine, and Vietnamese black glutinous rice wine. Chinese sweet rice wine (CSRW) is a traditional beverage consumed in China and prized by consumers for its unique flavor and purported nutritional properties related to its enrichment with oligosaccharides, polypeptides, vitamins, minerals, and amino acids [1]. CSRW is generally brewed with glutinous rice and has a low alcohol content, ranging from 0.5% to 14% (*v*/*v*), with a savory sweet taste that differs from Chinese yellow rice wine, which is brewed with rice or millet and has a higher alcohol content of 14–20% (*v*/*v*) [2,3]. In addition, CSRW fermentation also requires the addition of specific fermentation starters, also known as Jiuqu. Jiuqu likely originated over 5000 years ago in China, and records of its use are found in the QiMin YaoShu text written in the 6th century AD (ca. 544 AD) [1]. The Jiuqu for CSRW is made with rice flour and Chinese herbal medicine under open conditions, which is different from the wheat Qu (fermented by *Aspergillus*) used for Chinese yellow rice wine [4,5]. Regional variations in the wild native microbes and environmental conditions of Jiuqu production can potentially influence the microbial community composition and quality of Jiuqu, especially in non-sterile fermentation processes [6]. The influence of environment in different regions can also lead to differences in CSRW starters, which may impart distinct sensory profiles, flavors, and other characteristics to the CSRW [7].

Starters cultures are enriched with various microbes that participate in saccharification, fermentation, and the production of flavor-related metabolites, such as yeasts, filamentous fungi, and an array of bacterial taxa [8]. The microbial populations specific to different starters show variation in their metabolic capabilities that could determine the final flavor profile of Chinese rice wines [9]. For example, hydrolases, glucoamylases, proteases, esterases, lipases, and alcohol acetyl transferase are produced by various bacteria during fermentation to degrade the raw material substrates, all of which could lead to accumulation of aroma-related compounds or secondary metabolites and intermediates [10,11]. The fungal communities in starter cultures have also been shown to play an essential role in starch and protein hydrolysis, as well as in the production of ethanol, organic acids, higher alcohols, and esters [12].

Differences in raw materials can lead to differences in the number and diversity of microorganisms in starters [1,13,14]. Previous studies investigating the microbial diversity and volatile profiles of Chinese yellow rice wine starters have proposed that different microorganisms determine the different volatile compounds of the starter, which may affect the flavor of products. For instance, some studies have indicated that microbial heterogeneity in wheat Qu is responsible for significant differences in volatile compounds [5,13]. Another study found that bacterial and fungal communities varied significantly in different starters of Hongqu yellow rice wine (produced in Fujian, China) and that core microorganisms were positively correlated with specific organic acids and aromatic esters in the starters [14]. In addition, Chen et al. [15] reported that distinct microbial communities in three different traditional rice wine starters were associated with significantly different aroma components in the final fermented rice wines. One study comparing eight CSRW starter samples from different regions of southern China identified significant variation in the bacterial and fungal composition, which likely contributed substantial differences to the final flavor quality of the respective CSRWs [1]. While each of these studies provide some clues into the relationship between starter microbes and CSRW flavor, further detailed investigation of the metabolic characteristics, microbial composition, volatile compound profiles, and correlations between these factors in CSRW starters from different geographical regions are warranted to facilitate the development of defined starters that could improve the aromatic quality of CSRW. Furthermore, a more complete understanding of the microorganisms and their metabolic functions is needed to establish standardized strategies for industrial CSRW production.

In this paper, the composition, structure, diversity, and metabolic function of microbiota in CSRW starters from different geographic locations were compared using high-throughput metagenomic rDNA sequencing (i.e., 16S rRNA and ITS genes). In addition, we used gas chromatography–mass spectrometry to characterize volatile compounds in CSRW starters from different regions in order to examine whether and how different microbes in these CSRW starters might be correlated with specific aroma-related metabolites. Functional inference analysis was also conducted to compare enriched metabolic pathways in microbial communities of CSRW starters. Overall, correlations between microbes and aroma profiles from different regions, suggesting a link between microbiota function and flavor profiles associated with different CSRW starters, can improve our understanding of microbial community function and contribution to the flavor of traditional CSRW.

## 2. Materials and Methods

### 2.1. Sample Collection

**Figure 1:**
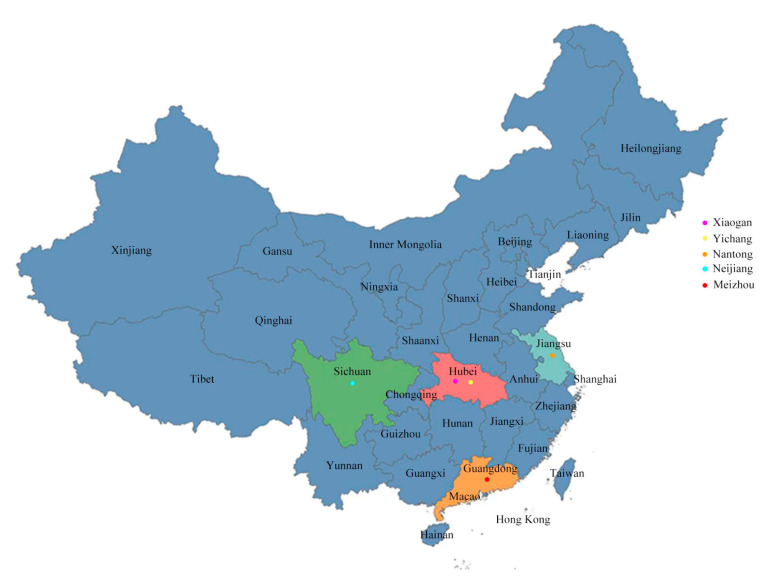
Samples from different parts of southern China.

Seven kinds of rice wine starters were sampled from a range of rural CSRW production regions in China, including YC1 and YC2 (Yichang, Hubei), XG (Xiaogan, Hubei), NT1 and NT2 (Nantong Jiangsu), NJ (Neijiang, Sichuan) and MZ (Meizhou, Guangdong) (Figure 1). The starter samples were delivered to the laboratory in a sterile sampling box containing ice bags and stored at −20 °C prior to GC-MS and sequencing analyses.

### 2.2. Volatile Compound Profile Analysis

Volatile compounds in rice wine starters and sweet rice wine samples were analyzed by headspace solid-phase microextraction coupled with gas chromatography–mass spectrometry (HS-SPME-GC-MS) [13]. Briefly, 0.8 g samples were mixed with 8 mL saturated NaCl solution in a 20 mL transparent round-bottom headspace sample bottle, to which was added 1 μL 2-octanol as the internal standard. For both wines, 1 µL 2-octanol internal standard and 2 g NaCl were added to 8 mL of each sample in a 20 mL vial. Carboxen/polydimethylsiloxane (CAR/PDMS) SPME fibers (75 μm, Fused Silica 24 Ga, Manual Holder, 3 pk, Supelco, St. Louis, MO, USA) were used for volatile compound extraction. The fiber was headed into the SPME device, which was inserted in the vial to extract volatile compounds at 60 °C for 50 min. Compounds were desorbed for 3 min at 280 °C in splitless mode, using a 0.75 mm dedicated SPME liner. The extraction of volatile compounds was carried out on an Agilent 7890 GC (Agilent Technologies, Santa Clara, CA, USA); equipped with a DB-5 capillary column: (60 m × 0.25 mm × 0.25 μm, Agilent Technologies, California, USA). The carrier gas was ultrahigh-purity helium at a constant flow of 1.0 mL/min [16]. Programmed temperature rise: initial temperature 50 °C, hold for 2 min; heated at 2 °C/min to 115 °C, hold for 3 min, 4 °C/min rise to 200 °C; 6 °C/min rise to 230 °C, hold for 10 min. Mass spectra (Agilent 5977B MS) were generated in the electron impact (EI) mode at 70 Ev ionization energy using the full scan mode (50–550 amu). Compounds were identified by comparison with the NIST11 mass spectral database. Semi-quantitation of the volatile compounds was achieved using 2-octanol as an internal standard and applying the formula C_2 = A_2/A_1 × C_1, where C_2 is the relative concentration of analyzed sample, C_1 is the final concentration of internal standard in sample, A_2 is the peak area of analyzed sample, and A_1 is the peak area of internal standard. The results were reported as the mean of three replicates of starter samples.

### 2.3. Bacterial and Fungal DNA Extraction and Sequencing

DNA was extracted from 0.3 g of starter samples using a PowerSoil Total DNA Isolation Kit (MO BIO, Carlsbad, CA, USA) according to manufacturer instructions. Extracts were made in triplicate and combined into a single extract of each sample. The concentration and quality of the extracted DNA were determined by spectrophotometry (NanoDrop 2000; Thermo, Japan) and 1% agarose gel electrophoresis. The 338F (5′-ACTCCTACGGGAGGCAGCAG-3′) and 806R (5′-GGACTACHVGGGTWTCTAAT-3′) primers were used to amplify the V3–V4 region of bacterial 16S rRNA genes [17]. The ITS1 (5′-CTTGGTCATTTAGAGGAAGTAA-3′) and ITS2 (5′-GCTGCGTTCTTCATCGATGC-3′) primers were used to amplify the ITS1 region of the fungal rRNA gene [18]. Libraries were prepared for high-throughput sequencing using a TruSeq^®^ DNA PCR-Free Sample Preparation Kit (Illumina, San Diego, CA, USA) and sequenced on the Illumina MiSeq PE300 platform by Majorbio Co., Ltd. (Shanghai, China), and the Illumina sequencing raw data were deposited in the Sequence Read Archive (SRA) in the National Center for Biotechnology Information (NCBI) database as a BioProject (https://www.ncbi.nlm.nih.gov/sra/PRJNA955836, accessed on 10 June 2023), accession number is PRJNA955836.

### 2.4. Bioinformatic Analysis

Raw pair-end reads were assembled after trimming adaptors and barcodes and filtering low-quality reads using Quantitative Insights into Microbial Ecology (QIIME) 1.9.1, resulting in clean, paired-end, high-quality reads. These sequences were then clustered into operational taxonomic units (OTUs) with 97% sequence similarity using Uparse software (version 7.1) and default parameters. The representative OTU sequences were also annotated using the Silva (version 138) and Unite (version 8.0) databases, respectively, with RDP-classifier (v.2.2) [16]. OTU abundance information was normalized using a standard sequence number corresponding to the sample with the fewest sequences to obtain the normalized data. Alpha and beta diversity metrics were calculated using QIIME 1.9.1. Phylogenetic investigation of communities by reconstruction of unobserved states 2 (PICRUSt2) was used to predict the functional of microbial communities based on the Green-genes database [19].

### 2.5. Statistical Analysis

Statistical calculations were performed using SPSS Statistics (v.22.0.0) software (IBM, Armonk, NY, USA). The volatile profiles were visualized with heatmap using R software (v.4.05) with the “heatmap” package. Differences in volatile metabolite profiles and beta diversity were visualized by principal component analysis (PCA) and principal coordinate analysis (PCoA) in R (v.4.05), respectively. The relative abundances of the representative taxa were visualized with R (v.4.05). To check the association of volatile flavor compounds with bacterial and fungal community composition, network analysis was performed using Spearman correlation analysis and visualized in the Gephi software (Version 0.8.2).

## 3. Results

### 3.1. Volatile Compounds in Different Starters

**Figure 2:**
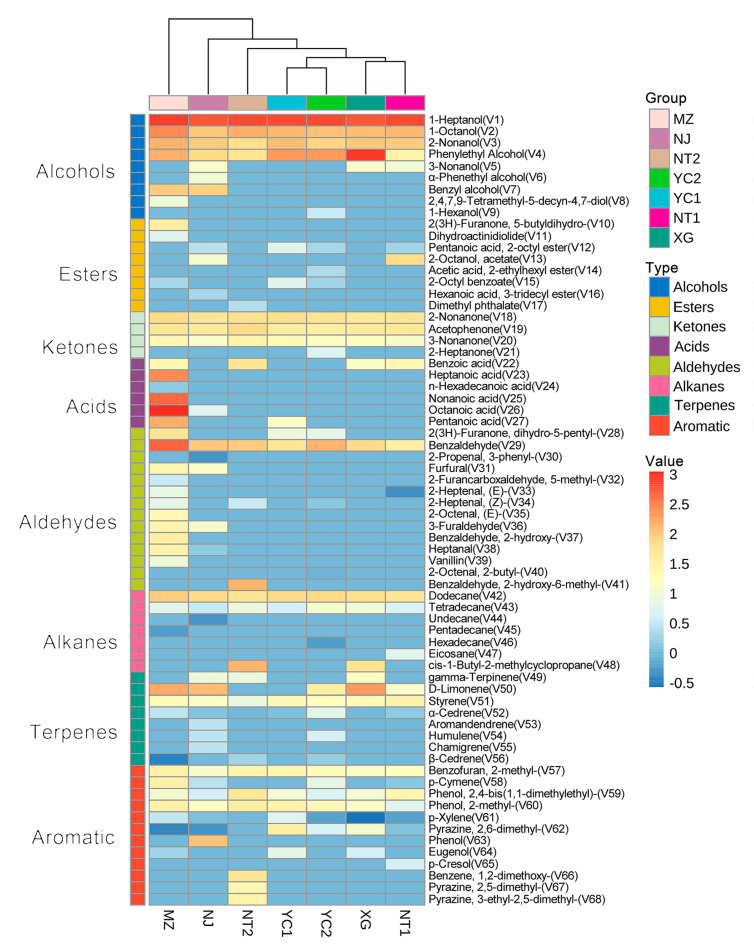
Heatmap of volatile compounds detected in CSRW starters. Legends show the log-transformed scores of their relative abundance.

The analysis of volatile compounds in the different traditional CSRW fermentation starters was performed by SPME-GC-MS. A combined total of 68 volatile compounds were identified in seven CSRW starters, mainly including nine alcohols, eight esters, six acids, fourteen aldehydes, four ketones, seven alkanes, eight terpenes, and twelve aromatic compounds; their detailed contents are listed in Supplementary Table S1, and the distribution of all of the volatile compounds is visualized in Figure 2. Close examination of the types and contents of volatiles in each sample (Supplementary Table S2) indicated that starters from MZ had the highest number of different volatile compound species as well as the highest content of all volatile compounds compared with other samples. Alcohols were the most abundant compounds and comprised the largest proportion of compounds detected in starters, accounting for 30.46–73.55% of samples. Among the different samples, XG had the highest alcohol content. In particular, phenylethyl alcohol was detected at relatively high levels in all samples. Acids (0–41.88%) and aldehydes (2.65–15.08%) were also relatively abundant among the volatile compounds in starters, with MZ samples having the highest content and types of acids and aldehydes. By contrast, ketones, esters, alkanes, terpenes, and aromatic compounds were relatively less abundant.

Furthermore, some volatile compounds were exclusively found in specific starter samples, such as 2-heptanone (V21), 2-butyl-2-octenal (V40), and hexadecane (V46) in YC2; eicosane (V47) and p-cresol (V65) in NT1; dimethyl phthalate (V17), 1,2-dimethoxy-benzene (V66), 2,5-dimethyl-Pyrazine (V67) and 3-ethyl-2,5-dimeth-yl-pyrazine (V68) in NT2; α-phenethyl alcohol (V6), 3-phenyl-2-propenal (V30), undecane (V44), aromandendrene (V53), chamigrene (V55), and phenol (V63) in NJ; and 2,4,7,9-tetramethyl-5-decyn-4,7-diol (V8), 5-butyldihydro-2(3H)-furanone (V10), dihydroactinidiolide (V11), heptanoic acid (V23), n-hexadecanoic acid (V24), nonanoic acid (V25), 5-methyl-2-furancarboxaldehyde (V32), (E)-2-octenal (V35), 2-hydroxy-benzaldehyde (V37), vanillin (V39), and pentadecane (V45) in MZ. By contrast, no volatile compounds were unique to XG or YC1 samples. Furthermore, 1-heptanol (V1), 1-octanol (V2), 2-nonanol (V3), phenylethyl alcohol (V4), 2-nonanone (V18), acetophenone (V19) and benzaldehyde (V29) were detected in all samples and at relatively high concentrations compared with other volatiles and were therefore considered the major volatile constituents of CSRW starters in this study. Some of these compounds were previously detected in CSRW [2,20]. Hierarchical clustering analysis of these samples indicated that YC1 and YC2 clustered together, and NT1 and XG clustered together, and that these two pairs of samples formed a group that clustered apart from NT2, NJ, and MZ samples. This clustering pattern indicated greater similarity in the volatile compound profiles among the former four samples than with those of the latter three samples.

**Figure 3:**
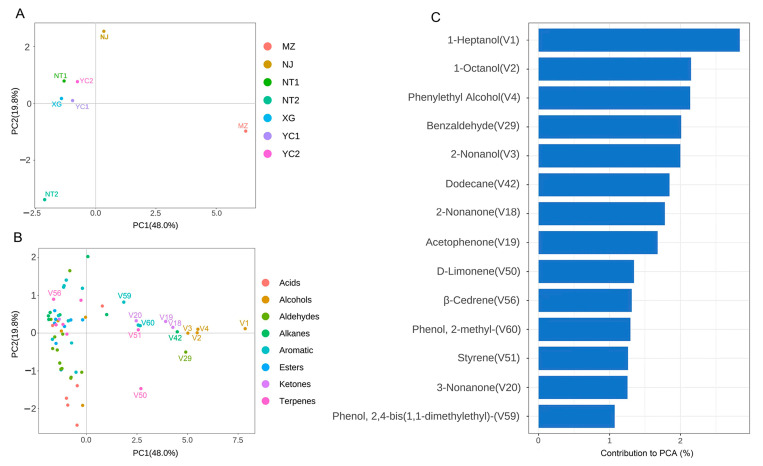
Variation in the volatile compounds of CSRW fermentation starter cultures from different geographic locations. (**A**) Bray–Curtis PCA plot of volatile compounds based on their relative contents. (**B**) PCA loading plot of the volatile compounds. (**C**) The volatile compounds significantly contributed to the observed variation in Bray–Curtis PCA plots.

A PCA distance plot of samples based on the relative contents of all of the volatile compound was used to visualize the variation among volatile profiles of different starters (Figure 3A), which was consistent with the hierarchical clustering analysis results mentioned above. The first principal component (PC1) accounted for 48.0% of the total variation, while PC2 explained 19.8%, collectively representing 67.8% of the total variability of volatile compounds. This result implied that the volatile profiles of YC1, YC2, NT1, and XG were more similar to each other (PC1 vs. PC2) than they were to the other three samples, and all grouped together in the second quadrant. In addition, NJ, NT2 and MZ exhibited little similarity to the other starters. These results indicated that samples from Yichang (YC1 and YC2) and Xiaogan (XG), two cities in Hubei Province, have similar aroma characteristics, while the samples from Nantong city (NT1 and NT2) in Jiangsu Province showed marked differences. In addition, these results identify differences in the aroma characteristics of samples from different regions, which could potentially lead to variation in the aroma profiles of the final products, making each CSRW regionally distinct, as proposed by Su and co-workers [7]. A loading plot of the 68 compounds is shown in Figure 3B. The 14 main contributors (contribution > 1%) to regional variation in volatiles were 1-heptanol (V1), 1-octanol (V2), phenylethyl alcohol (V4), benzaldehyde (V29), 2-nonanol (V3), dodecane (V42), 2-nonanone (V18), acetophenone (V19), d-limonene (V50), β-Cedrene (V56), 2-methyl-phenol (V60), styrene (V51), 3-nonanone (V20), and 2,4-bis(1,1-dimethylethyl)-phenol (V59) (Figure 3C).

### 3.2. Alpha Diversity Analysis of Different Starters

In order to assess the microbial diversity in CSRW starters, trimmed and filtered sequencing data were pooled and reads were clustered at a 97% similarity level, resulting in a total of 1110 bacterial OTUs, and 103 fungal OTUs. Good’s coverage was higher than 99.7% for each sample, indicating that the sequencing depth was sufficient to reliably describe the full diversity of microbial communities. Chao1, ACE, Shannon, and Simpson indices were calculated to characterize the α-diversity of microbiota in each starter sample (Supplementary Table S3).

The Chao1 and ACE indices show the abundance of microbial communities, with higher scores indicating higher total abundance of OTUs, which differed between bacteria and fungi. For bacterial OTUs, Chao1 and ACE revealed that NT1 and NT2 had greater microbial community richness, followed closely by YC1, XG and YC2. For fungal OTUs, YC1 had the most abundant microbial community, followed by NT2 and NT1. The Shannon and Simpson indices reflect the diversity of the microbial community, with higher Shannon scores and lower Simpson scores indicating higher microbial community diversity, which also differed among bacteria and fungi. For bacterial OTUs, NT2 starter samples had the highest microbial diversity, while YC2 had the lowest microbial diversity. Among fungal OTUs, YC1 had the highest diversity, whereas YC2, XG, and MZ had lower diversity. In general, NT2 had greater bacterial community richness and higher bacterial community diversity, while YC1 had greater fungal community richness and diversity.

### 3.3. Beta Diversity Analysis of Different Starters

**Figure 4:**
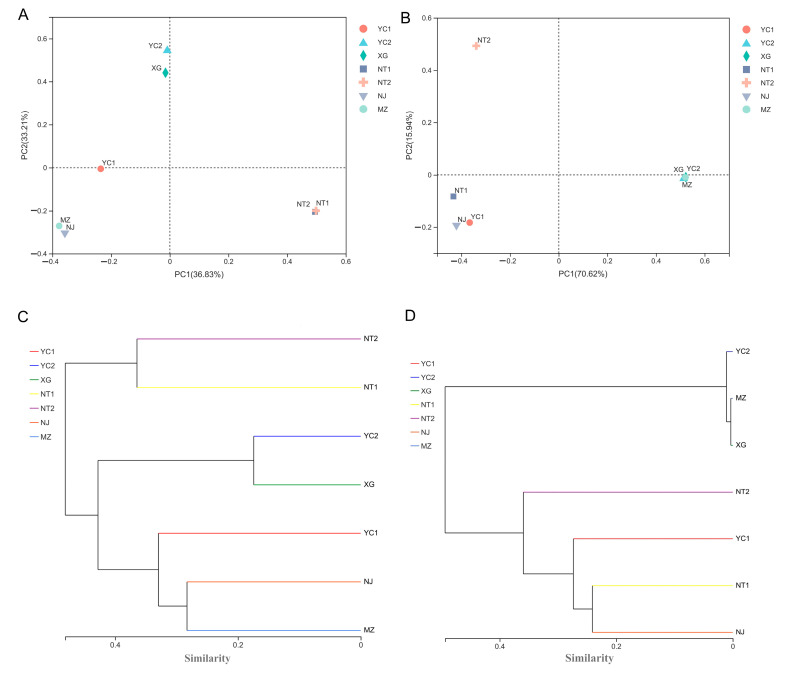
The beta diversity (PCoA) of microbial communities of CSRW starter samples from different geographic locations. (**A**) PCoA of bacterial communities within starter samples; (**B**) PCoA of fungal communities within starter samples; and (**C**) hierarchical cluster analysis of bacterial communities and (**D**) fungal communities.

To analyze the beta diversity of bacteria and fungi in the samples of starters from different regions, microbial community profiles were compared by generating Bray–Curtis PCoA plots. In the bacterial community profiles (Figure 4A), PC1 and PC2 accounted for 36.83% and 33.21% of the observed variability, respectively, collectively explaining 70.04% of the total variability of the bacterial community. The resulting PCoA plots revealed that bacterial communities in starter samples clustered into four distinct groups, with XG and YC2 in quadrant 2, YC1 on the horizontal ordinate, NJ and MZ in quadrant 3, NT1 and NT2 in quadrant 4. This result highlighted the effects of differences in CSRW starters in different regions.

Principal coordinate plots of fungal communities in these samples indicated that PC1 and PC2 explained 70.62% and 15.94% of the observed variability, respectively, collectively representing 86.56% of the total variability among starter fungal communities (Figure 4B). By contrast with bacterial communities, the fungal communities clustered into only three different groups by quadrant, with NT2 in quadrant 2; NJ, YC1, and NT1 (which had relatively similar fungal distributions) forming a group in quadrant 3; and XG, YC2, and MZ samples clustered in quadrant 4, which differed slightly from the results of the bacteria. Hierarchical clustering of the CSRW starter samples based on the abundance of bacterial and fungal genera showed similar results to the PCoA plot (Figure 4C,D). Taken together, these results showed that the microbial communities of CSRW starters from different regions exhibited both variability and similarities, which was consistent with findings in previous analyses of CSRW starters [1,6]. The close position among samples indicated similarities in the composition of principal genera, while more distant samples in the PCoA plot reflected differences in these principal genera. Fungal communities in these samples showed large differences in PC1 values, indicating their large differences in principal genera. These findings led us to further explore the differences in microbial community composition among samples.

### 3.4. Microbial Composition and Core Microbiota of Starters

#### 3.4.1. Bacterial Composition

**Figure 5:**
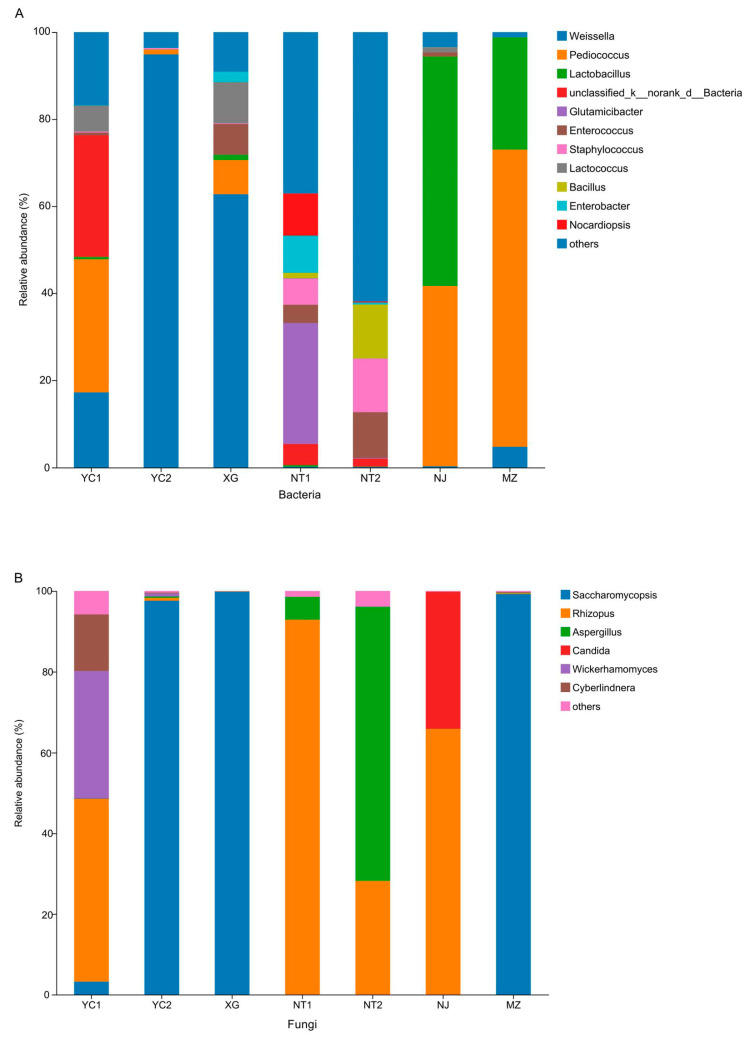
Genus-level relative abundance of microorganisms (>1%) in CSRW starter samples. Bacteria (**A**), Fungi (**B**).

A total of 594 bacterial genera were detected in seven starter samples, of which 11 had a relative abundance greater than 1%, including *Weissella* (0.13–94.87%), *Pediococcus* (0–68.25%), *Lactobacillus* (0.06–52.81%), unclassified_k__norank_d__Bacteria (0–27.93%), *Glutamicibacter* (0–27.74%), *Enterococcus* (0–10.50%), *Staphylococcus* (0–12.28%), *Lactococcus* (0–9.44%), *Bacillus* (0–12.43%), *Enterobacter* (0–8.53%), and *Nocardiopsis* (0–9.70%) (Figure 5A). *Weissella*, which was the most abundant genus in CSRW starters, had a higher abundance in YC1, YC2, and XG, and was especially characteristic of YC2 and XG. Other LAB genera were also abundant in samples, including *Pediococcus*, *Lactobacillus*, *Lactococcus*, and *Enterococcus*. Among these, *Pediococcus* was more abundant in MZ, NJ, and YC1 than other the samples, and was the characteristic genus in MZ and YC1, although this genus was undetectable in NT1. *Lactobacillus* and *Lactococcus* each accounted for a large proportion of bacterial genera in the starter samples. In particular, *Lactobacillus* was found at its highest relative abundance in NJ, while *Lactococcus* was most abundant in XG compared to the other samples. In addition, *Enterococcus* was found in high levels in NT2, NT1, and XG samples.

The predominant genus in NT1 samples was *Glutamicibacter*. Among other prevalent genera, *Staphylococcus* and *Enterobacter* were found mainly in NT1 and NT2 samples, while *Bacillus* was detected at particularly high levels in NT2. A less abundant genus, *Nocardiopsis*, was largely found in NT1 samples.

#### 3.4.2. Fungal composition

Analysis of fungal taxa revealed a total of 58 genera in the seven combined starter samples. Among these, six genera had a relative abundance higher than 1%, including *Saccharomycopsis* (0–99.84%), *Rhizopus* (0.11–92.86%), *Aspergillus* (0–67.94%), *Candida* (0–33.91%), *Wickerhamomyces* (0–31.52%), and *Cyberlindnera* (0–14.03%) (Figure 5B). *Saccharomycopsis* accounted for a considerable proportion of fungi in MZ, YC2, and XG samples. However, this genus was not detected in NT1 and NT2 samples, suggesting that, despite its prevalence in some samples, *Saccharomycopsis* was not ubiquitously present in CSRW starters. In contrast with *Saccharomycopsis*, *Rhizopus* was found in high abundance in NT1, NJ, YC1, and NT2 samples and was the only core fungus present in all samples. *Aspergillus* also accounted for a considerable proportion of fungi in these samples, particularly in NT2. Compared with the above-mentioned microbes, *Candida*, *Wickerhamomyces*, and *Cyberlindnera* occupied a relatively smaller proportion of fungi. *Candida* was found primarily in the NJ samples, while *Wickerhamomyces* was more abundant in YC1. *Cyberlindnera* was prevalent in the YC1 sample.

### 3.5. Microbial Function Prediction

**Figure 6:**
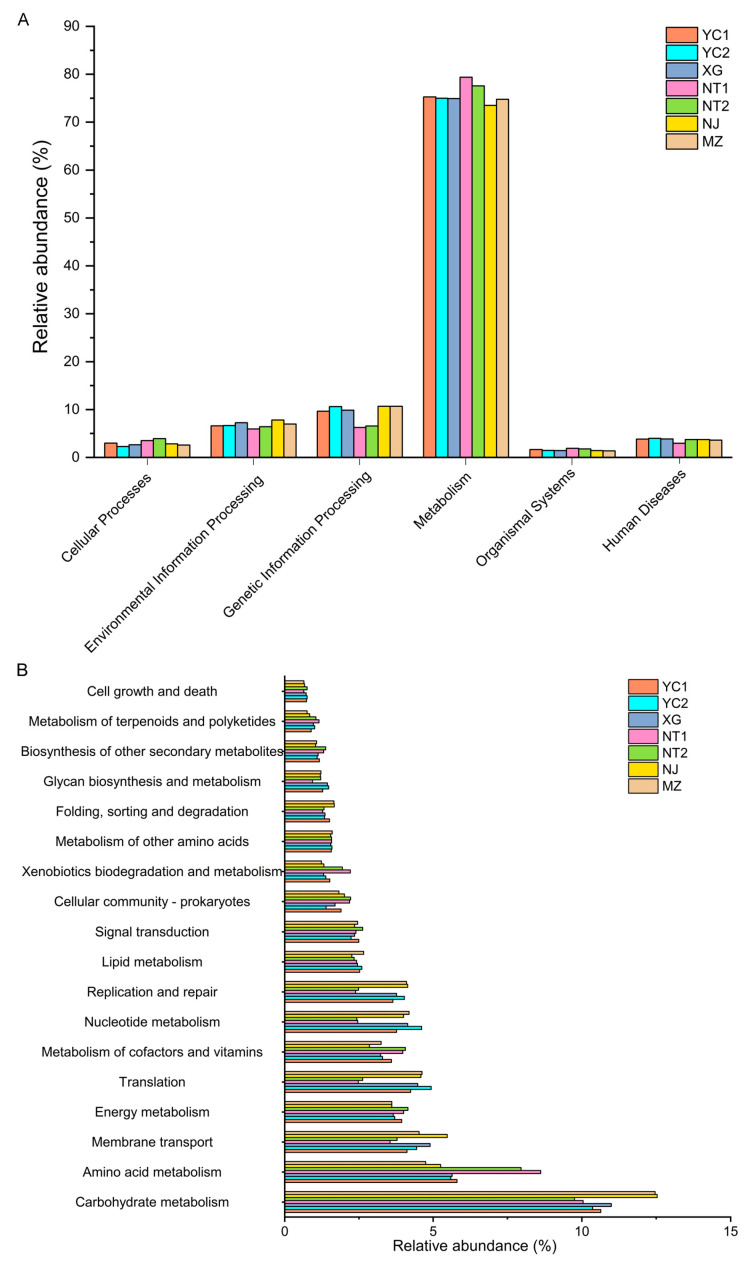
Predicted functional profiles of bacterial communities in fermentation starters from different regions. (**A**) Bar graph of level 1 KEGG functional categories inferred by PICRUSt2. (**B**) Bar graph of level 2 KEGG functional categories inferred by PICRUSt2.

To better understand how different taxa in starters may contribute to the specific flavor properties of CSRW, PICRUSt2 (phylogenetic investigation of communities by reconstruction of unobserved states 2) analysis [21] was conducted to infer the functional metabolic capabilities of microbial communities based on their composition in 16s rRNA gene metagenomic data in different starters [15]. The predicted functional genes enriched in the CSRW starters were related to metabolism (73.51–79.39%), genetic information processing (6.27–10.68%), environmental information processing (5.95–7.83%), human diseases (2.96–3.99%), cellular processes (2.28–3.93%), and organismal systems (1.38–1.91%) among the level 1 KEGG pathways (Figure 6A). Metabolism-related pathways were markedly enriched in most samples, especially NT1, and NT1 samples had a relatively low abundance of predicted genes related to human disease. Among the level 2 KEGG pathway categories, the most abundant predicted metabolic capabilities were related to carbohydrate metabolism, followed by amino acid metabolism, membrane transport, and energy metabolism (Figure 6B). Inferred carbohydrate metabolism pathways were prominent in MZ and NJ samples while amino acid metabolism pathways were prominent in NT1 and NT2 samples, which may have contributed to the observed variation in volatile compound profiles between these samples.

### 3.6. Correlation Analysis between Volatile Components and Representative Microbiota

**Figure 7:**
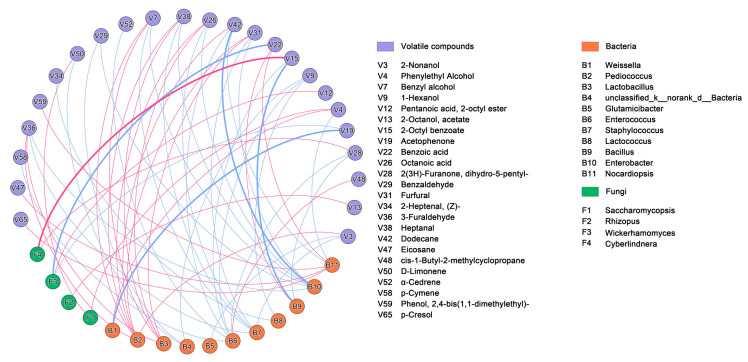
Co-occurrence analysis based on Spearman correlations between all of the volatile components of starters and predominant microbial genera. All correlations had *p* value < 0.05 and |r| > 0.7. Red lines represent positive correlations between volatile components and microorganisms, blue lines represent negative correlations between volatile components and microorganisms. Bold connecting lines represent highly significant correlations (|r| > 0.7, *p* < 0.01).

In order identify significant relationships between specific microbial taxa and the production of specific flavor compounds, a Spearman correlation analysis was conducted for the predominant bacterial (n = 11, relative content > 1%) and fungal (n = 6, relative content > 1%) genera and the full set of detected volatile compounds. This analysis yielded a total of 57 significant pair-wise correlations (|r| > 0.7, *p* < 0.05) between 24 different volatiles and 15 microbial genera, including 25 positive correlations (red lines) and 32 negative correlations (blue lines). Among these correlations, one pair of highly significant positive correlations and four pairs of highly significant negative correlations (|r| > 0.7, *p* < 0.01) emerged (Figure 7).

In particular, an important positive correlation was observed between *Weissella* and phenylethyl alcohol (V4), while this genus negatively correlated with acetophenone (V19) (*p* < 0.01). *Pedioccoccus* was positively correlated with 2-nonanol (V3), benzyl alcohol (V7), octanoic acid (V26), furfural (V31), 3-furaldehyde (V36), heptanal (V38), and dodecane (V42). In addition, *Lactobacillus* had similar positive correlations with the same volatile components as *Pedioccoccus*. The relatively low-abundance genus, *Nocardiopsis*, was positively correlated with some volatile compounds such as benzoic acid (V22), eicosane (V47), 2,4-bis(1,1-dimethylethyl)-phenol (V59), and p-cresol (V65). *Enterococcus* was positively correlated with cis-1-Butyl-2-methylcyclopropane (V48). *Bacillus* was positively correlated with benzoic acid (V22) and was significantly negatively correlated with 2-octyl benzoate (V15) (*p* < 0.01). *Enterobacter* shared a highly significant negative correlation with dodecane (V42) (*p* < 0.01), while *Glutamicibacter* was negatively correlated with 2-nonanol (V3). Among fungi, *Saccharomycopsis* was positively correlated with phenylethyl alcohol (V4) and d-limonene (V50); *Rhizopus* was positively correlated with 2-octanolacetate (V13); *Wickerhamomyces* was positively correlated with 2-octyl ester pentanoic acid (V12), but was negatively correlated with benzoic acid (V22) (*p* < 0.01); *Cyberlindnera* shared a highly significant positive correlation with 2-octyl benzoate (V15) (*p* < 0.01) and was positively correlated with dihydro-5-pentyl-2(3H)-furanone (28).

In general, bacteria had 19 positive pair-wise associations with volatile components, while fungal taxa shared 6 positive pair-wise associations, suggesting that bacteria may exert somewhat greater influence on the aroma profiles of CSRW starters, and which is consistent with previous findings in Wheat Qu [13]. In addition, the most important positive correlations between two major volatile compounds and three dominated microbial genera, including *Weissella* and *Saccharomycopsis* and phenylethyl alcohol, *Pediococcus* and 2-nonanol. This study showed that alcohols, acids, and aldehydes account for the largest proportion of volatile compounds in starter samples, in addition, esters were also the primary volatile compounds in Chinese rice wine [22]. Based on their significant correlation with these important volatile compounds, the five bacterial genera (including *Weissella*, *Pediococcus*, *Lactobacillus*, *Bacillus* and *Nocardiopsis*) and four fungal genera (including *Saccharomycopsis*, *Rhizopus*, *Wickerhamomyces*, and *Cyberlindnera*) were considered the core functional microorganisms in CSRW starters.

### 3.7. Relationship between CSRW and Starter Cultures in Volatile Compounds

In order to understand the relationship and difference between aroma substances in CSRWs and in CSRW starters, and better understand the factors that lead to the formation of aroma in CSRWs, lay a foundation for later research on the relationship between microbes of CSRW starters and aroma in CSRWs, we determined the aroma of CSRWs. The research results show that a combined total of 85 volatile compounds were identified in the seven CSRWs (Supplementary Table S4), mainly including thirteen alcohols, twenty-seven esters, ten acids, seven aldehydes, one ketone, twelve alkanes, five terpenes, and ten aromatic compounds. Compared with the starter samples, CSRWs had a greater number of different alcohols, esters, organic acids, and alkanes, with esters found in notably greater relative abundance in CSRWs than starters. While starters from MZ have the widest variety of volatile metabolites, CSRWs from YC1 and YC2 reportedly have more complex aroma profiles. Similarly, starter samples from MZ have the highest contents of aromatic compounds, and CSRW from NJ have higher aromatic compound contents than those from MZ, indicating that both CSRW and starters are distinct between regions, and that the content and diversity of metabolites in starters do not necessarily determine the aroma profiles their respective fermentation products. Twenty-three volatile compounds overlapped between the CSRW samples and starters (Supplementary Table S5), among which alcohols and alkanes comprised the largest proportion. In addition, some of the predominant volatile compounds found in relatively high concentration in CSRWs were also present in starters, including 1-heptanol, 1-octanol, 2-nonanol, phenylethyl alcohol, 2-nonanone, and benzaldehyde. Among these identified volatile compounds, phenylethyl alcohol and benzaldehyde had markedly higher contents in CSRWs than in starters (Supplementary Tables S1 and S2).

## 4. Discussion

In this study, we characterized microbiota and volatile compounds and explore correlations between them associated with fermentation starters for Chinese sweet rice wine from seven regions across China. Through GC-MS analysis, we found that alcohols, acids, and aldehydes are the main aroma substances, accounting for a high proportion. Alcohols are the main aroma components in brewed rice wine and higher alcohols make a great contribution to the smell and taste and provide the alcohol precursor required for the synthesis of desirable esters in rice wine [22]. Volatile organic acids are known to contribute to distinctive flavors in rice wine, with aromas described as acidic, or similar to cheese and sweat, while aldehyde volatiles impart floral and nutty qualities to rice wine [22,23]. Esters were relatively less abundant in CSRW starters, although esters related to floral and fruity flavors have been proved to be the main aroma substances of rice wine, which may be due to esters produced by microbial metabolism during fermentation and a series of reactions of chemical substances in wine [14,16]. In addition, we have reported 1-heptanol, 1-octanol, 2-nonanol, phenylethyl alcohol, 2-nonanone, acetophenone, and benzaldehyde as the predominant volatile compounds among a wide range of potential flavor-related metabolites. Among the predominant organic volatiles, the higher alcohols 1-heptanol (V1), 1-octanol (V2), 2-nonanol (V3), and phenylethyl alcohol (V4) were the major quantitative components of rice wine, mainly contributing a unique flavor of sweet, floral, fruit and rose aroma, according to previous studies [9,24]. 2-nonanone (V18), as a common aroma in rice wine, reportedly contributes flower, fruit and herb aromas, acetophenone (V19) contributed a flowery aroma, and benzaldehyde (V29), an important aldehyde substance in rice wine, contributed almond and burnt sugar aromas [2]. Some of these compounds were previously detected in CSRW, implying that these volatile compounds in starters may be further produced during fermentation, and contribute to the aroma profile of the final rice wine. Furthermore, the large majority of these compounds have been identified in starters for different types of wine, such as wheat Qu, used to start Chinese yellow rice wine, and Hong Qu, the fermentation starter for Hongqu yellow rice wine [13,14]. It is worth noting that 2-methyl-phenol (V60) and 2,4-bis(1,1-dimethylethyl)-phenol (V59) were unique volatile compounds in this study and have not been previously reported in other fermentation starters.

Metagenomic 16s rRNA gene analysis indicated that the bacterial composition was relatively variable among the regional starter samples, which is in agreement with previous findings that wheat Qu for yellow rice wine and Da Qu starters for Chinese liquor production also show high regional variability [13,25]. Despite the variability, these starter samples were generally dominated by lactic acid bacteria (LAB). As purported probiotics, LAB are considered beneficial to human health. The majority of LAB genera, especially *Lactobacillus*, which were found to be positively correlated with 2-nonanol, benzyl alcohol, octanoic acid, furfural, 3-furaldehyde, heptanal, and dodecane in our study, can produce lactic acid and a variety of antimicrobial agents to exclude pathogens, and thus enhance the relatively safety of fermentation environments during the brewing process [26]. Moreover, LAB play critical roles in the production of proteins and metabolites such as esterases, lipases, and alcohol acetyl transferases that reportedly contribute to the formation of flavor-related components of wine [10]. Among the prevalent LAB genera, *Weissella* showed the highest abundance, especially in YC1, YC2, and XG starters, consistent with previous studies that found *Weissella* is the dominant genus in rice wine koji [6], although other studies have identified *Bacillus* as a dominant genus among starter microbiota [1]. In addition, *Weissella* is a representative genus in CSRW starters of Hubei province, which was consistent with previous studies that showed *Weissella* was a representative genus in Wheat Qu of Hubei province [13]. As lactic acid bacteria (LAB), *Weissella* can produce short-chain fatty acids and esters during food fermentation [26,27]. In this study, an important positive correlation was observed between *Weissella* and phenylethyl alcohol, supporting that *Weissella* has been shown to play an important role in the flavor properties of rice wine [28]. By contrast, MZ and YC1 samples were characterized by high levels of *Pediococcus*, which is commonly identified as a core microbe in starters for different sweet rice wines [1,4], although it was undetectable in some samples in the current study (i.e., NT1). The correlation analysis showed that *Pedioccoccus* was positively correlated with some main volatile compounds, including alcohols, acids, and aldehydes, which provides a new reference for the bacteria to improve the aroma of rice wine, and it was reported that *Pediococcus* can increase the content of organic acids, short-chain fatty acids and esters during food fermentation [27,29]. The result supports the conclusion that *Pediococcus* enhanced the production of volatile acids, alcohols, and esters in Shaoxing-jiu [28]. *Pediococcus* can also reportedly stabilize fermentation communities against food spoilage bacteria and pathogens [29]. *Enterococcus* was positively correlated with cis-1-Butyl-2-methylcyclopropane. Research showed that *Enterococcus* was regarded as one of the major flavor producers in the fermentation of food [30]. These collective results supported the likelihood that lactic acid bacteria can promote the accumulation of flavor substances during food fermentation [26]. The predominant genus in NT1 samples was *Glutamicibacter*, which was previously shown to be associated with chili pepper [31]. Given that this is a plant-associated genus, this discovery might reflect the addition of the Chinese herbal medicine lalaocao, similar to chili pepper, in starter samples. *Staphylococcus* and *Enterobacter*, also frequently identified among rice wine microbiota, were also found mainly in NT1 and NT2 samples [5,30], both of which can pose a threat to food safety and human health in some contexts [1]. *Bacillus* was found in particularly high levels in NT2 and was positively correlated with benzoic acid. This finding is unsurprising, given the important role of this genus in the fermentation of several foods through extensive secretion of hydrolytic enzymes such as amylases, acid proteases, and fibrinolytic enzymes, and this activity may also facilitate the formation of flavor-related volatile metabolites in rice wine [11]. Moreover, *Bacillus* can directly produce a broad range of volatile compounds such as pyrazines, aldehydes, ketones, and alcohols [1]. A less abundant genus, *Nocardiopsis*, was largely found in NT1 samples, but has been rarely reported in other studies.

For fungi, our study showed that *Saccharomycopsis* accounted for a considerable proportion in starter samples. This non-*Saccharomyces* yeast is frequently identified in starter samples and rice wines with varying abundance [30] and is present in the fermentation starters of various foods [32]. In addition, *Saccharomycopsis* can secrete amylase, protease, and β-glucosidase, which contributes to saccharifying starches during fermentation [33]. The correlation analysis revealed that *Saccharomycopsis* was positively correlated with phenylethyl alcohol and d-limonene and a previous study found that non-*Saccharomyces* yeast of *Saccharomycopsis* could regulate alcohol content and enhance the wine aromatic quality, by co-fermenting with *Candida* [14]. However, this genus was not detected in NT1 and NT2 samples, suggesting that despite its prevalence in some samples, *Saccharomycopsis* is not ubiquitously present in CSRW starters. In our study, *Rhizopus* was the only core fungus present in all samples. Due to its high capacity for amylase and glucoamylase production, *Rhizopus* was commonly found in various amylolytic fermentation starters, and made a great contribution to the flavor formation of wine and can decompose starch into glucose, subsequently producing lactic acid and alcohol [28]. *Rhizopus* is well-known to substantially contribute to metabolite production in rice wine [30,34]. In addition, our study showed that *Rhizopus* was positively correlated with 2-octanolacetate and this result proved the conclusion that *Rhizopus* can produce many hydrolytic enzymes and flavoring compounds, including esters [1]. Among the fungal taxa, *Aspergillus* is a well-studied genus of filamentous fungi that can reportedly affect flavor by producing various hydrolytic enzymes (including amylases, glucoamylases, proteases, lipases, and xylanases) that hydrolyze starch, protein and lipid macromolecules into dextrin, maltose, glucose, small peptides, and fatty acids, which are then consumed as a growth substrate by yeasts and other microorganisms [35]. *Candida* and *Wickerhamomyces* are both non-*Saccharomyces* yeasts, and previous studies have shown that such yeasts may play a prominent role in the production of secondary metabolites that influence the flavor and sensory profiles of wine [36]. Co-fermentation of non-*Saccharomyces* yeast with *Saccharomyces* can potentially improve both alcohol content and the aromatic qualities of wine [37]. In our study, the correlation analysis showed that *Wickerhamomyces* was positively correlated with pentanoic acid, 2-octyl ester, but was negatively correlated with benzoic acid and *Wickerhamomyces* has been frequently isolated from wines and their secondary metabolites are related to the taste and flavor of wine [14,16]. *Cyberlindnera* was prevalent in the YC1 sample, and our study showed that *Cyberlindnera* shared positive correlation with 2-octyl benzoate and dihydro-5-pentyl-2(3H)-furanone. Previous studies have shown that *Cyberlindnera* is positively correlated with β-glucosidase content and increased aroma compounds in tea [38].

In the current work, inferred function analyses supported enrichment for carbohydrate and amino acid metabolism pathways in these starters, implying that flavor formation in these samples was likely linked to protein and starch metabolism [13,15]. The main metabolic pathways varied in different starter samples, which may have contributed to the observed variation in volatile compound profiles between these samples. The analysis of CSRWs from different regions showed that esters were the most important aroma substances, supporting that esters are the main and characteristic substances in rice wine [16]; relatively few esters are detectable in CSRW starters, suggesting that ester formation is likely attributable to microbial metabolism during the fermentation and aging processes. In addition, twenty-three volatile compounds were present in both starter samples and CSRWs samples; these results indicated that the aroma-related compounds in starters were retained in the final CSRW products, and possibly further produced during fermentation, suggesting a starter-specific contribution to the final aroma profile, as proposed in previous studies [39]. Among these identified volatile compounds, phenylethyl alcohol and benzaldehyde had markedly higher contents in CSRWs than in starters (Supplementary Tables S1 and S2), supporting the likelihood that these two predominant volatiles did not solely originate in the starters, but were produced by metabolic processes during fermentation. Overall, the aroma profiles of the CSRWs were more complex, and characterizing their constituent volatile compounds can improve our understanding of the factors underlying aroma quality and facilitate the exploration of the mechanisms responsible for shaping aroma profiles. In addition, these findings provide a basis for future targeted studies of the relationship between CSRW aromas and specific taxa in starters. Moreover, based on the results, better starter cultures may be developed.

## 5. Conclusions

This study explores the volatile compounds, microbial diversity, and their relationship in CSRW starters from different geographic regions of China. A total of 68 volatile flavor-related compounds were detected. *Weissella*, *Pediococcus* and *Lactobacillus* were dominant bacterial genera, while *Saccharomycopsis* and *Rhizopus* were dominant among fungal genera in CSRW starters. Correlation analysis revealed 15 important volatiles were significantly positively correlated with nine microbial genera, including five bacterial genera (i.e., *Weissella*, *Pediococcus*, *Lactobacillus*, *Enterococcus*, *Bacillus*, and *Nocardiopsis*) and four fungal genera (i.e., *Saccharomycopsis*, *Rhizopus*, *Wickerhamomyces*, and *Cyberlindnera*) (*p* < 0.05, |r| >0.7), and these microorganisms were considered the core functional microorganisms in CSRW starters. The most important positive correlations were detected between phenylethyl alcohol and *Weissella* or *Saccharomycopsis* and between 2-nonanol and *Pediococcus*. Twenty-three volatile compounds overlapped between CSRWs and starters, suggesting that starters contribute to shaping the final aroma profile of CSRWs. This study can facilitate the isolation and culture of these taxa for the development of defined CSRW starters for targeted flavor profiles in future work.

## Data Availability

Data is contained within the article.

## Supplementary Materials

The following supporting information can be downloaded at: https://www.mdpi.com/article/10.3390/foods12152932/s1, Table S1: Relative content of volatile compounds within Chinese sweet rice wine starters; Table S2: Volatile compounds of type and content (µg/L) within Chinese sweet rice wine starters; Table S3: α-diversity of the bacteria and fungus community of starters; Table S4: Relative content of volatile compounds within Chinese sweet rice wine samples; Table S5: The same volatile compounds of Chinese sweet rice wine samples and starter samples.

## References

[1] Cai H., Zhang T., Zhang Q., Luo J., Cai C., Mao J. (2018). Microbial diversity and chemical analysis of the starters used in traditional Chinese sweet rice wine. Food Microbiol..

[2] Yang Y., Zhong H., Yang N., Zhu D., Li J., Yang Z. (2022). Effects of the proteins of indica rice and indica waxy rice on the formation of volatiles of sweet rice wine. Int. J. Food Sci. Technol..

[3] Zhong J., Ye X., Fang Z., Xie G., Liao N., Shu J. (2012). Determination of biogenic amines in semi-dry and semi-sweet Chinese rice wines from the Shaoxing region. Food Control.

[4] Zhao X., Wang Y., Cai W., Yang M., Zhong X., Guo Z., Shan C. (2020). High-throughput sequencing-based analysis of microbial diversity in rice wine koji from different areas. Curr. Microbiol..

[5] Yang H., Peng Q., Zhang H., Sun J., Shen C., Han X. (2022). The volatile profiles and microbiota structures of the wheat Qus used as traditional fermentation starters of Chinese rice wine from Shaoxing region. LWT.

[6] Zhao X., Shan C., Cai W., Guo Z., Hou Q., Yang X. (2022). Bacterial community structure and prediction of microbial metabolic pathway in rice wine koji from different regions, a traditional fermented food in China. Front. Microbiol..

[7] Su Y.T., Zhao S.M. (2014). Research progress of process technology and quality characteristics of sweet rice wine. China Brew..

[8] He F., Lin X., Tong Z. (2015). Production Process and Technology of Yellow Rice Wine.

[9] Yang Y., Xia Y., Wang G., Yu J., Ai L. (2017). Effect of mixed yeast starter on volatile flavor compounds in Chinese rice wine during different brewing stages. LWT.

[10] Gammacurta M., Lytra G., Marchal A., Marchand S., Barbe J.C., Moine V. (2018). Influence of lactic acid bacteria strains on ester concentrations in red wines: Specific impact on branched hydroxylated compounds. Food Chem..

[11] Simonen M., Palva I. (1993). Protein secretion in *Bacillus* species. Microbiol. Rev..

[12] Medina K., Boido E., Fariña L., Gioia O., Gomez M.E., Barquet M. (2013). Increased flavour diversity of Chardonnay wines by spontaneous fermentation and co-fermentation with *Hanseniaspora vineae*. Food Chem..

[13] Xiao C., Wang L., Zhang Y.G., Tu T.Y., Wang S.T., Shen C.H.A. (2021). Comparison of microbial communities and volatile compounds in wheat Qu from different geographic locations. LWT.

[14] Huang Z.R., Guo W.L., Zhou W.B., Li L., Xu J.X., Hong J.L. (2019). Microbial communities and volatile metabolites in different traditional fermentation starters used for Hong Qu glutinous rice wine. Food Res. Int..

[15] Chen L., Ren L., Li D., Ma X. (2021). Analysis of microbiomes in three traditional starters and volatile components of the Chinese rice wines. Food Sci. Biotechnol..

[16] Liu Z., Wang Z., Sun J., Ni L. (2020). The dynamics of volatile compounds and their correlation with the microbial succession during the traditional solid-state fermentation of Gutian Hong Qu glutinous rice wine. Food Microbiol..

[17] Du R., Wu Q., Xu Y. (2020). Chinese liquor fermentation: Identification of key flavor-producing *Lactobacillus* spp. by quantitative profiling with indigenous internalstandards. Appl. Environ. Microbiol..

[18] Ji Z., Jin J., Yu G., Mou R., Mao J., Liu S. (2018). Characteristic of filamentous fungal diversity and dynamics associated with wheat Qu and the traditional fermentation of Chinese rice wine. Int. J. Food Sci. Technol..

[19] Langille M.G., Zaneveld J., Caporaso J.G., McDonald D., Knights D., Reyes J.A. (2013). Predictive functional profiling of microbial communities using 16S rRNA marker gene sequences. Nat. Biotechnol..

[20] Yang Y., Zhong H., Yang N., Xu S., Yang T. (2022). Quality improvement of sweet rice wine fermented with *Rhizopus delemar* on key aroma compounds content, phenolic composition, and antioxidant capacity compared to *Rhizopus oryzae*. J. Food Sci. Technol..

[21] Deng L., Mao X., Liu D., Ning X.Q., Shen Y., Chen B. (2020). Comparative analysis of physicochemical properties and microbial composition in high-temperature Daqu with different colors. Front. Microbiol..

[22] Yu H., Xie T., Xie J., Ai L., Tian H. (2019). Characterization of key aroma compounds in Chinese rice wine using gas chromatography-mass spectrometry and gas chromatography–olfactometry. Food Chem..

[23] Chen S., Wang C., Qian M., Li Z., Xu Y. (2019). Characterization of the key aroma compounds in aged Chinese rice wine by comparative aroma extract dilution analysis, quantitative measurements, aroma recombination, and omission studies. J. Agric. Food Chem..

[24] Yang Y., Zhong H., Yang T., Lan C., Zhu H. (2021). Characterization of the key aroma compounds of a sweet rice alcoholic beverage fermented with *Saccharomycopsis fibuligera*. J. Food Sci. Technol..

[25] Zheng X.W., Yan Z., Robert Nout M.J., Boekhout T., Han B.Z., Zwietering M.H. (2015). Characterization of the microbial community in different types of Daqu samples as revealed by 16S rRNA and 26S rRNA gene clone libraries. World J. Microbiol. Biotechnol..

[26] Cappello M.S., Zapparoli G., Logrieco A., Bartowsky E.J. (2017). Linking wine lactic acid bacteria diversity with wine aroma and flavour. Int. J. Food Microbiol..

[27] Kamboj K., Vasquez A., Balada-Llasat J.M. (2015). Identification and significance of *Weissella* species infections. Front. Microbiol..

[28] Chen C., Liu Y., Tian H., Ai L., Yu H. (2020). Metagenomic analysis reveals the impact of JIUYAO microbial diversity on fermentation and the volatile profile of Shaoxing-jiu. Food Microbiol..

[29] Porto M.C.W., Kuniyoshi T.M., Azevedo P.O.S., Vitolo M., Oliveira R.S. (2017). *Pediococcus* spp.: An important genus of lactic acid bacteria and pediocin producers. Biotechnol. Adv..

[30] Zhao C., Su W., Mu Y., Jiang L., Mu Y. (2020). Correlations between microbiota with physicochemical properties and volatile flavor components in black glutinous rice wine fermentation. Food Res. Int..

[31] Xu X., Wu B., Zhao W., Lao F., Chen F., Liao X. (2021). Shifts in autochthonous microbial diversity and volatile metabolites during the fermentation of chili pepper (*Capsicum frutescens* L.). Food Chem..

[32] Seo S.H., Park S.E., Yoo S.A., Lee K.I., Na C.S., Son H.S. (2016). Metabolite profiling of Makgeolli for the understanding of yeast fermentation characteristics during fermentation and aging. Process Biochem..

[33] Chi Z., Chi Z., Liu G., Wang F., Ju L., Zhang T. (2009). *Saccharomycopsis fibuligera* and its applications in biotechnology. Biotechnol. Adv..

[34] Liu S., Yang L., Zhou Y., He S., Li J., Sun H. (2019). Effect of mixed moulds starters on volatile flavor compounds in rice wine. LWT.

[35] Lv X.C., Cai Q.Q., Ke X.X., Chen F., Rao P.F., Ni L. (2015). Characterization of fungal community and dynamics during the traditional brewing of Wuyi Hong Qu glutinous rice wine by means of multiple culture-independent methods. Food Control.

[36] Liu S., Laaksonen O., Kortesniemi M., Kalpio M., Yang B. (2018). Chemical composition of bilberry wine fermented with non-*Saccharomyces* yeasts (*Torulaspora delbrueckii* and *Schizosaccharomyces pombe*) and *Saccharomyces cerevisiae* in pure, sequential and mixed fermentations. Food Chem..

[37] Englezos V., Rantsiou K., Cravero F., Torchio F., Ortizjulien A., Gerbi V. (2016). *Starmerella bacillaris* and *Saccharomyces cerevisiae* mixed fermentations toreduce ethanol content in wine. Appl. Microbiol. Biotechnol..

[38] Wang R., Sun J., Lassabliere B., Yu B., Liu S.Q. (2015). β-Glucosidase activity of *Cyberlindnera* (*Williopsis*) *saturnus* var. mrakii NCYC 2251 and its fermentation effect on green tea aroma compounds. LWT.

[39] Yuan G.Y., He Y.L., Wang C.X., Qiu S.Y. (2022). Research progress on the factors correlated with flavor quality in rice wine. Food Ferment. Ind..

