# Supplementary material for: Microbial Communities and Correlation between Microbiota and Volatile Compounds in Fermentation Starters of Chinese Sweet Rice Wine from Different Regions
Source: Foods. 2023 Aug 2;12(15):2932. doi: 10.3390/foods12152932 (PMC10419015; doi:10.3390/foods12152932)
Supplement: Supplementary file 1 [file foods-12-02932-s001.zip › foods-2520167-supplementary.pdf]

Supplementary Material

1 Supplementary Tables

Supplementary Table S1 Relative content of volatile compounds within Chinese sweet rice wine starters.

No.	Compound	Classification	Relative content (μg/L)						
			YC1	YC2	XG	NT1	NT2	NJ	MZ
V1	1-Heptanol	Alcohols	714.91	682.10	622.94	688.89	686.89	601.68	815.89
V2	1-Octanol		131.65	112.78	119.04	133.68	156.64	107.44	276.95
V3	2-Nonanol		126.41	82.53	105.31	94.09	67.19	97.45	144.95
V4	Phenylethyl Alcohol		227.21	218.06	892.11	30.47	57.50	72.94	159.91
V5	3-Nonanol		0.00	0.00	13.00	10.20	0.00	13.94	0.00
V6	α-Phenethylalcohol		0.00	0.00	0.00	0.00	0.00	10.48	0.00
V7	Benzylalcohol		0.00	0.00	0.00	0.00	0.00	91.60	96.69
V8	2,4,7,9-Tetramethyl-5-decyn-4,7-diol		0.00	0.00	0.00	0.00	0.00	0.00	9.09
V9	1-Hexanol		0.00	3.49	0.00	0.00	0.00	0.00	1.11
V10	2(3H)-Furanone,5-butyldihydro-	Esters	0.00	0.00	0.00	0.00	0.00	0.00	37.08
V11	Dihydroactinidiolide		0.00	0.00	0.00	0.00	0.00	0.00	5.64
V12	Pentanoicacid,2-octylester		5.60	2.12	0.00	1.90	0.00	2.78	0.00
V13	2-Octanol,acetate		0.00	0.00	0.00	69.42	0.00	11.01	0.00
V14	Aceticacid,2-ethylhexylester		0.00	2.32	0.00	0.00	0.00	0.00	0.00
V15	2-Octylbenzoate		5.60	1.86	0.00	0.00	0.00	0.00	2.02
V16	Hexanoicacid,3-tridecylester		0.00	0.00	0.00	0.00	0.00	2.13	0.00
V17	Dimethyl phthalate		0.00	0.00	0.00	0.00	2.42	0.00	0.00

V18	2-Nonanone	Ketones	69.66	60.78	51.03	52.00	62.34	53.28	77.88
V19	Acetophenone		41.24	34.38	40.88	50.67	77.01	45.02	55.74
V20	3-Nonanone		19.59	15.54	15.08	13.54	29.86	13.78	22.69
V21	2-Heptanone		0.00	4.73	0.00	0.00	0.00	0.00	0.00
V22	Benzoic acid	Acids	0.00	0.00	14.50	24.30	52.87	0.00	23.08
V23	Heptanoic acid		0.00	0.00	0.00	0.00	0.00	0.00	288.73
V24	n-Hexadecanoicacid		0.00	0.00	0.00	0.00	0.00	0.00	1.55
V25	Nonanoicacid		0.00	0.00	0.00	0.00	0.00	0.00	472.60
V26	Octanoicacid		0.00	0.00	0.00	0.00	0.00	5.20	1124.08
V27	Pentanoicacid		14.00	0.00	0.00	0.00	0.00	0.00	158.86
V28	2(3H)-Furanone,dihydro-5-pentyl-	Aldehydes	8.24	6.86	0.00	0.00	0.00	0.00	56.87
V29	Benzaldehyde		55.58	149.12	77.13	35.82	98.41	100.33	520.64
V30	2-Propenal,3-phenyl-		0.00	0.00	0.00	0.00	0.00	0.49	0.00
V31	Furfural		0.00	0.00	0.00	0.00	0.00	14.16	24.70
V32	2-Furancarboxaldehyde,5-methyl-		0.00	0.00	0.00	0.00	0.00	0.00	3.51
V33	2-Heptenal,(E)-		0.00	0.00	0.00	0.45	0.00	0.00	7.02
V34	2-Heptenal,(Z)-		0.00	1.42	0.00	0.00	3.32	0.00	6.76
V35	2-Octenal,(E)-		0.00	0.00	0.00	0.00	0.00	0.00	23.03
V36	3-Furaldehyde		0.00	0.00	0.00	0.00	0.00	12.01	28.30
V37	Benzaldehyde,2-hydroxy-		0.00	0.00	0.00	0.00	0.00	0.00	35.28
V38	Heptanal		0.00	0.00	0.00	0.00	0.00	1.45	29.65
V39	Vanillin		0.00	0.00	0.00	0.00	0.00	0.00	9.06
V40	2-Octenal,2-butyl-		0.00	0.91	0.00	0.00	0.00	0.00	0.00
V41	Benzaldehyde,2-hydroxy-6-methyl-		0.00	0.00	0.00	0.00	140.13	0.00	0.00
V42	Dodecane	Alkanes	72.03	74.24	69.14	53.65	58.11	73.05	99.52

V43	Tetradecane		4.12	11.52	10.38	4.75	8.70	3.49	6.14
V44	Undecane		0.00	0.00	0.00	0.00	0.00	0.49	0.00
V45	Pentadecane		0.00	0.00	0.00	0.00	0.00	0.00	0.61
V46	Hexadecane		0.00	0.59	0.00	0.00	0.00	0.00	0.00
V47	Eicosane		0.00	0.00	0.00	5.52	0.00	0.00	0.00
V48	cis-1-Butyl-2-methylcyclopropane		0.00	0.00	56.82	0.00	141.04	0.00	0.00
V49	gamma-Terpinene		0.00	0.00	17.07	0.00	7.75	9.02	0.00
V50	D-Limonene		0.00	35.69	226.06	14.43	0.00	125.02	173.00
V51	Styrene		22.53	17.62	20.79	29.88	8.20	14.92	21.66
V52	α-Cedrene	Terpenes	1.12	6.04	0.00	1.51	0.00	1.27	2.92
V53	Aromandendrene		0.00	0.00	0.00	0.00	0.00	3.00	0.00
V54	Humulene		0.00	4.17	0.00	0.00	0.00	2.81	0.00
V55	Chamigrene		0.00	0.00	0.00	0.00	0.00	2.83	0.00
V56	β-Cedrene		0.00	1.68	0.00	0.00	1.84	0.00	0.36
V57	Benzofuran,2-methyl-		25.01	19.65	14.59	16.93	28.24	13.33	33.45
V58	p-Cymene		0.00	6.64	0.00	1.37	0.00	2.69	33.41
V59	Phenol,2,4-bis(1,1-dimethylethyl)-		10.33	4.84	10.23	24.81	49.89	4.05	12.38
V60	Phenol,2-methyl-		29.04	24.01	13.76	5.83	40.01	15.94	31.50
V61	p-Xylene		5.04	0.63	0.27	0.67	0.00	1.12	3.20
V62	Pyrazine,2,6-dimethyl-	Aromatic	35.94	4.52	11.86	1.23	0.00	0.49	0.40
V63	Phenol		0.00	0.00	0.00	0.00	0.00	118.39	0.00
V64	Eugenol		6.86	0.00	3.92	0.00	0.00	0.00	1.94
V65	p-Cresol		0.00	0.00	0.00	4.13	0.00	0.00	0.00
V66	Benzene,1,2-dimethoxy-		0.00	0.00	0.00	0.00	53.42	0.00	0.00
V67	Pyrazine,2,5-dimethyl-		0.00	0.00	0.00	0.00	26.26	0.00	0.00

V68	Pyrazine,3-ethyl-2,5-dimethyl-	0.00	0.00	0.00	0.00	30.31	0.00	0.00

Supplementary Table S2 Volatile compounds of type and content (μg/L) within Chinese sweet rice wine starters.

**Sample**		**YC1**	**YC2**	**XG**	**NT1**	**NT2**	**NJ**	**MZ**
**Alcohols**	**Type**	4	5	5	5	4	7	7
	**Content**	1200.18	1098.96	1752.4	957.33	968.22	995.53	1504.59
**Esters**	**Type**	2	3	0	2	1	3	3
	**Content**	11.2	6.3	0	71.31	2.42	15.92	44.74
**Ketones**	**Type**	3	4	3	3	3	3	3
	**Content**	130.5	115.44	106.99	116.21	169.2	112.08	156.31
**Acids**	**Type**	1	0	1	1	1	1	6
	**Content**	14	0	14.5	24.3	52.87	5.2	2068.9
**Aldehydes**	**Type**	2	4	1	2	3	5	11
	**Content**	63.81	158.31	77.13	36.26	241.86	128.44	744.8
**Alkanes**	**Type**	2	3	3	3	3	3	3
	**Content**	76.15	86.35	136.34	63.92	207.85	77.03	106.26
**Terpenes**	**Type**	2	5	3	3	3	7	4
	**Content**	23.65	65.2	263.91	45.81	17.79	158.87	197.94
**Aromatic**	**Type**	6	6	6	7	6	7	7
	**Content**	112.21	60.29	54.63	54.97	228.14	156.02	116.29
**Total**	**Type**	22	30	22	26	24	36	44
	**Content**	1631.7	1590.85	2405.9	1370.11	1888.35	1649.09	4939.83

Supplementary Table S3 α-diversity of the bacteria and fungus community of starters.

Sample	Goods coverage		OTU number		Chao1		ACE		Shannon		Simpson	
	Bacteria	Fungi	Bacteria	Fungi	Bacteria	Fungi	Bacteria	Fungi	Bacteria	Fungi	Bacteria	Fungi
**YC1**	1	1	137	54	138.43	54.55	138.54	56.3	2.15	1.63	0.21	0.28
**YC2**	0.999	1	46	22	81	25.33	141.26	26.83	0.31	0.16	0.9	0.95
**XG**	0.999	1	88	14	119.23	19.25	123.4	22.56	1.49	0.01	0.41	1
**NT1**	0.997	1	791	32	836.99	33.67	843.27	36.36	3.73	1.04	0.1	0.44
**NT2**	0.999	1	516	38	528.2	47.33	529.21	45.6	4.16	1.04	0.05	0.48
**NJ**	1	1	45	20	54.43	29.33	57.85	44.02	1.09	0.66	0.43	0.55
**MZ**	1	1	26	18	39.75	19.5	69.93	19.97	1.05	0.06	0.5	0.98

Note: The first column is the sample name, and the remaining columns are the corresponding values of the diversity index type in each sample

Supplementary Table S4. Relative content of volatile compounds within Chinese sweet rice wine samples.

No.	Compound	Classification	Relative content (μg/L)						
			YC1	YC2	XG	NT1	NT2	NJ	MZ
V1	1-Butanol, 2-methyl-		0.00	2467.00	536.05	0.00	0.00	0.00	0.00
V2	1-Butanol, 3-methyl-		0.00	7564.02	7109.39	7762.41	1378.94	0.00	10970.49
V3	1-Heptanol		0.00	170.68	160.05	155.56	97.83	135.75	293.45
V4	1-Octanol		242.49	246.62	231.22	105.51	126.86	669.75	0.00
V5	1-Propanol, 2-methyl-		0.00	2442.14	1276.53	2425.31	0.00	0.00	0.00
V6	1-Propanol, 3-ethoxy-		0.00	67.99	30.49	0.00	0.00	93.25	0.00
V7	2,3-Butanediol	Alcohols	3651.70	871.28	385.23	304.44	38.28	277.57	855.92
V8	2,3-Butanediol, [R-(R*,R*)]-		0.00	0.00	0.00	0.00	0.00	1949.29	0.00
V9	2-Nonanol		0.00	127.44	60.64	90.55	126.93	0.00	126.65
V10	Ethanol		7000.00	9562.38	8500.46	12908.45	12046.61	14840.04	10876.95
V11	Phenylethyl Alcohol		54.49	9982.77	5825.28	4845.54	4728.99	140.06	3860.55
V12	a-Cadinol		45.42	0.00	0.00	0.00	0.00	0.00	0.00
V13	1-Hexanol		0.00	82.65	0.00	0.00	0.00	0.00	0.00
V14	2-Propenoic acid, 3-phenyl-, ethyl ester		0.00	0.00	50.16	0.00	0.00	46.14	0.00
V15	Acetic acid, 2-phenylethyl ester		147.07	0.00	128.20	0.00	299.92	323.05	0.00
V16	Benzeneacetic acid, ethyl ester		360.45	139.52	53.35	33.78	89.21	203.30	102.33
V17	Benzoic acid, ethyl ester	Esters	217.97	112.25	98.36	56.42	137.31	0.00	144.80
V18	Butanedioic acid, diethyl ester		3106.50	2483.10	468.57	624.15	386.76	464.17	549.47
V19	Butanedioic acid, ethyl 3-methylbutyl ester		111.31	54.43	0.00	0.00	0.00	98.41	0.00

V20	Decanedioic acid, diethyl ester		132.70	26.77	0.00	0.00	0.00	0.00	0.00
V21	Decanoic acid, ethyl ester		1465.40	1060.73	736.33	453.39	1506.68	3177.18	220.75
V22	Diethyl suberate		201.01	62.23	60.09	0.00	7.95	64.37	0.00
V23	Dimethyl phthalate		0.00	20.21	10.02	0.00	40.85	0.00	60.95
V24	Dodecanoic acid, ethyl ester		1409.20	779.29	334.29	472.38	896.48	3233.42	0.00
V25	E-11-Hexadecenoic acid, ethyl ester		346.87	0.00	0.00	82.94	0.00	0.00	0.00
V26	Ethyl 9-hexadecenoate		3168.42	121.07	386.20	1201.23	356.58	2169.55	0.00
V27	Ethyl Acetate		0.00	1094.52	0.00	0.00	0.00	0.00	0.00
V28	Heptadecanoic acid, ethyl ester		144.12	0.00	0.00	48.62	35.96	95.90	0.00
V29	Hexadecanoic acid, ethyl ester		16543.07	6227.64	12074.03	19026.56	19626.69	39879.65	1546.72
V30	Hexadecanoic acid, methyl ester		45.54	0.00	0.00	0.00	0.00	56.72	0.00
V31	Hexanoic acid, ethyl ester		0.00	0.00	0.00	0.00	98.40	0.00	0.00
V32	Nonanoic acid, 9-oxo-, ethyl ester		392.05	94.65	0.00	0.00	0.00	0.00	0.00
V33	Nonanoic acid, ethyl ester		170.63	109.04	38.56	0.00	94.28	75.71	58.06
V34	Octanoic acid, ethyl ester		383.64	400.64	288.09	219.01	723.35	653.18	183.90
V35	Pentadecanoic acid, 3-methylbutyl ester		0.00	0.00	0.00	0.00	0.00	83.20	0.00
V36	Pentadecanoic acid, ethyl ester		230.70	46.79	20.35	83.67	141.99	397.23	0.00
V37	Tetradecanoic acid, ethyl ester		2552.94	960.30	809.54	1915.99	2180.34	7973.75	246.67
V38	1,4-Benzenedicarboxylic acid, diethyl ester		29.38	0.00	0.00	0.00	0.00	0.00	0.00
V39	Hexadecanoic acid, propyl ester		30.06	0.00	0.00	0.00	0.00	0.00	0.00
V40	Isoamyl laurate		0.00	0.00	0.00	0.00	0.00	62.00	0.00
V41	2-Nonanone	Ketones	0.00	39.03	23.11	12.44	35.73	0.00	47.65
V42	Acetic acid	Acids	1934.50	1061.11	1023.07	1217.33	0.00	0.00	1257.66
V43	Benzoic acid		0.00	173.13	0.00	51.28	150.24	0.00	0.00

V44	cis-9-Hexadecenoic acid		0.00	0.00	0.00	0.00	0.00	81.78	0.00
V45	n-Decanoic acid		394.76	129.96	0.00	0.00	0.00	0.00	0.00
V46	n-Hexadecanoic acid		587.99	43.85	0.00	0.00	0.00	147.98	0.00
V47	Dodecanoic acid		80.15	0.00	0.00	0.00	0.00	0.00	0.00
V48	Gamolenic Acid		1101.97	0.00	0.00	0.00	0.00	0.00	0.00
V49	Nonanoic acid		174.31	0.00	0.00	0.00	0.00	0.00	0.00
V50	Oleic Acid		176.22	0.00	0.00	0.00	0.00	0.00	0.00
V51	Tetradecanoic acid		170.78	0.00	0.00	0.00	0.00	0.00	0.00
V52	9,17-Octadecadienal, (Z)-		9504.53	663.18	0.00	2928.55	1541.29	10716.06	0.00
V53	Benzaldehyde		214.03	579.54	320.86	102.77	422.85	426.01	2746.02
V54	Benzeneacetaldehyde		429.25	433.85	300.90	127.19	260.17	610.27	306.01
V55	Benzeneacetaldehyde, .alpha.-ethylidene-	Aldehydes	0.00	104.21	0.00	0.00	0.00	0.00	0.00
V56	Diethyl pimelate		0.00	20.26	0.00	0.00	0.00	0.00	0.00
V57	Furfural		63.04	337.86	305.92	181.76	858.93	351.67	617.46
V58	Nonanal		0.00	0.00	0.00	0.00	0.00	0.00	120.30
V59	Benzene, hexyl-		683.66	507.99	107.00	262.88	729.49	1187.11	315.27
V60	Butane, 1,1-diethoxy-3-methyl-		0.00	0.00	0.00	0.00	0.00	27.28	0.00
V61	cis-1-Butyl-2-methylcyclopropane		0.00	0.00	0.00	0.00	0.00	0.00	103.54
V62	Dodecane		723.34	616.10	301.81	324.34	606.92	1143.17	340.21
V63	Eicosane	Alkanes	61.08	0.00	0.00	84.49	59.71	14.99	0.00
V64	Hexadecane		462.67	112.02	97.43	187.21	430.74	281.13	157.26
V65	Octadecane		174.89	38.46	0.00	140.06	166.33	129.79	0.00
V66	Pentadecane		0.00	39.57	0.00	36.15	127.04	123.51	0.00
V67	Tetradecane		444.07	140.55	109.08	121.61	381.08	259.51	237.15

V68	trans-1-Butyl-2-methylcyclopropane		0.00	0.00	0.00	0.00	0.00	0.00	74.34
V69	Tridecane		16118.54	13668.92	6549.02	8510.98	12956.33	24562.92	6469.62
V70	Hexacosane		337.99	0.00	0.00	0.00	0.00	0.00	0.00
V71	α-Cedrene		0.00	0.00	0.00	0.00	0.00	0.00	182.46
V72	1-Octene		0.00	0.00	0.00	39.05	33.83	0.00	24.10
V73	Caryophyllene	Terpenes	0.00	0.00	80.64	0.00	0.00	109.17	0.00
V74	D-Limonene		38.55	33.45	45.09	50.43	49.56	85.42	39.01
V75	Humulene		193.85	47.21	32.84	0.00	0.00	72.34	0.00
V76	Biphenyl		1575.05	117.58	108.60	2040.49	306.84	662.98	1813.12
V77	Oxime-, methoxy-phenyl-_		268.09	0.00	0.00	183.84	199.06	0.00	0.00
V78	Phenol		109.43	38.26	0.00	29.94	34.58	0.00	0.00
V79	Phenol, 2,4-bis(1,1-dimethylethyl)-		0.00	249.67	0.00	0.00	0.00	0.00	0.00
V80	Phenol, 4-ethyl-	Aromatic	0.00	213.03	20.75	53.48	167.11	0.00	147.87
V81	Phthalic anhydride		718.19	0.00	0.00	0.00	0.00	0.00	0.00
V82	2-Methoxy-4-vinylphenol		152.47	104.55	50.96	66.77	104.67	239.61	90.85
V83	Naphthalene		61.84	207.67	0.00	0.00	0.00	0.00	0.00
V84	Phenol, 4-ethyl-2-methoxy-		0.00	0.00	0.00	0.00	0.00	0.00	31.43
V85	Pyrazine, tetramethyl-		1575.05	117.58	108.60	2040.49	306.84	662.98	1813.12

Supplementary Table S5. The same volatile compounds of Chinese sweet rice wine samples and starter samples.

No.	Compound	Classification
V1	1-Heptanol	
V2	1-Octanol	
V3	2-Nonanol	Alcohols
V4	Phenylethyl Alcohol	
V5	1-Hexanol	
V6	Dimethyl phthalate	Esters
V7	2-Nonanone	Ketones
V8	Benzoic acid	
V9	n-Hexadecanoic acid	Acids
V10	Nonanoic acid	
V11	Benzaldehyde	Aldehydes
V12	Furfural	
V13	cis-1-Butyl-2-methylcyclopropane	
V14	Dodecane	
V15	Eicosane	
V16	Hexadecane	Alkanes
V17	Octadecane	
V18	Pentadecane	
V19	Tetradecane	
V20	D-Limonene	Terpenes
V21	Humulene	
V22	Phenol	Aromatic
V23	Phenol, 2,4-bis(1,1-dimethylethyl)-	
